# Barriers to Initiation of Antiretrovirals during Antituberculosis Therapy in Africa

**DOI:** 10.1371/journal.pone.0019484

**Published:** 2011-05-12

**Authors:** Dominique J. Pepper, Suzaan Marais, Robert J. Wilkinson, Feriyl Bhaijee, Virginia De Azevedo, Graeme Meintjes

**Affiliations:** 1 Institute of Infectious Diseases and Molecular Medicine, University of Cape Town, Cape Town, South Africa; 2 Infectious Diseases Unit, GF Jooste Hospital, Cape Town, South Africa; 3 Department of Medicine, University of Cape Town, Cape Town, South Africa; 4 Division of Medicine, Imperial College London, United Kingdom; 5 MRC National Institute for Medical Research, Mill Hill, London, United Kingdom; 6 City Health, Cape Town, South Africa; Fundació Institut Germans Trias i Pujol; Universitat Autònoma de Barcelona CibeRES, Spain

## Abstract

**Background:**

In the developing world, the principal cause of death among HIV-infected patients is tuberculosis (TB). The initiation of antiretroviral therapy (ART) during TB therapy significantly improves survival, however it is not known which barriers prevent eligible TB patients from initiating life-saving ART.

**Method:**

*Setting.* A South African township clinic with integrated tuberculosis and HIV services. *Design.* Logistic regression analyses of a prospective cohort of HIV-1 infected adults (≥18 years) who commenced TB therapy, were eligible for ART, and were followed for 6 months.

**Findings:**

Of 100 HIV-1 infected adults eligible for ART during TB therapy, 90 TB patients presented to an ART clinic for assessment, 66 TB patients initiated ART, and 15 TB patients died. 34% of eligible TB patients (95%CI: 25–43%) did not initiate ART. Male gender and younger age (<36 years) were associated with failure to initiate ART (adjusted odds ratios of 3.7 [95%CI: 1.25–10.95] and 3.3 [95%CI: 1.12–9.69], respectively). Death during TB therapy was associated with a CD4+ count <100 cells/µL.

**Conclusion:**

In a clinic with integrated services for tuberculosis and HIV, one-third of eligible TB patients – particularly young men – did not initiate ART. Strategies are needed to promote ART initiation during TB therapy, especially among young men.

## Introduction

Tuberculosis (TB) is frequently encountered in South Africa and is associated with considerable morbidity and mortality. In 2007, the estimated TB incidence in South Africa was 948/100,000 people in the general population[1] – the fifth highest in the world[2]. In the same year, it is estimated that there were 461,000 new TB cases, and that 112,000 TB deaths occurred [1]. Co-infection with human immunodeficiency virus type-1 (HIV-1) is responsible for this high mortality[3]: In South Africa, co-infection occurs in 73% of people diagnosed with TB and 84% of people who die with TB[1].

Restoring immune function with antiretroviral treatment (ART) can reduce the high morbidity and mortality of TB. While considerable debate exists whether ART should be commenced early or late *during* TB treatment, there is compelling evidence that ART in eligible patients should not be deferred until after TB treatment[4].

The provision of ART to eligible TB patients during TB treatment is clearly a priority for South Africa[5]. While little is known about obstacles to ART initiation during TB treatment, data exists for HIV/AIDS cohorts. Losina *et al* report that almost 50% of patients in their cohort did not have a CD4+ count within 8 weeks of HIV diagnosis [6]. Moreover, up to 2 months may elapse between obtaining the initial CD4 count and first ART training (7). These delays are concerning, especially as a substantial proportion of HIV-related morbidity and mortality occurs after 2 months of TB therapy[4], [8].

Timely initiation of ART requires efficient assessment of eligible TB patients, as well as elimination of barriers to ART initiation. Here, we performed a secondary analysis of a recently-described prospective cohort of HIV-1 infected TB patients[8] in order to determine the barriers to ART initiation.

## Methods

### Study population

We conducted our study in a high density (>7500 inhabitants/km^2^), predominantly black township in South Africa [9], where TB case notification rates approach 1,600/100,000 people of the general population annually. TB patients in this township are treated in TB clinics administered by Cape Town's Health Department. According to national protocol, TB patients receive standardized TB treatment regimens using Directly Observed Therapy Short-course (DOTS)[10]. National guidelines recommended ART for all TB patients with a CD4+ cell count less than 200 cells/µL or a history of a WHO stage 4 illness[10]. Extra-pulmonary tuberculosis – although a WHO stage 4 illness – was *not* an indication for ART unless the patient's CD4+ count was less than 200 cells/µL. First-line ART during our study was stavudine, lamivudine, and either nevirapine or efavirenz. Efavirenz was preferred for adults receiving rifampin-based TB treatment. National guidelines also recommended daily trimethoprim-sulfamethoxazole (160/800 mg) chemoprophylaxis[9].

Our study center is one of the first in South Africa to successfully integrate ART and TB health-care services. As a result, our TB cohort is characterised by high rates of i) voluntary counselling and testing of HIV status (>95%), ii) rigorous testing of CD4+ counts if HIV-infected (≈99%), and iii) provision of trimethoprim-sulfamethoxazole chemoprophylaxis (>95%)[8]. Moreover, DOTS coverage is >80% at this center (personal communication –Judy Caldwell, Cape Town Health Department).

We have previously described our prospective cohort of 209 HIV-infected TB patients (≥18 years of age), which were recruited at our study center. Data obtained from this cohort was used to determine the incidence, risk factors and causes of clinical deterioration during 6 months of TB therapy[8]. All adults in our cohort were recruited at initiation of TB therapy – regardless of HIV status – and followed for 6 months. Written informed consent was obtained from enrolled adults and the Research Ethics Committee of the University of Cape Town approved this study (REC 178/2008).

**Figure 1:**
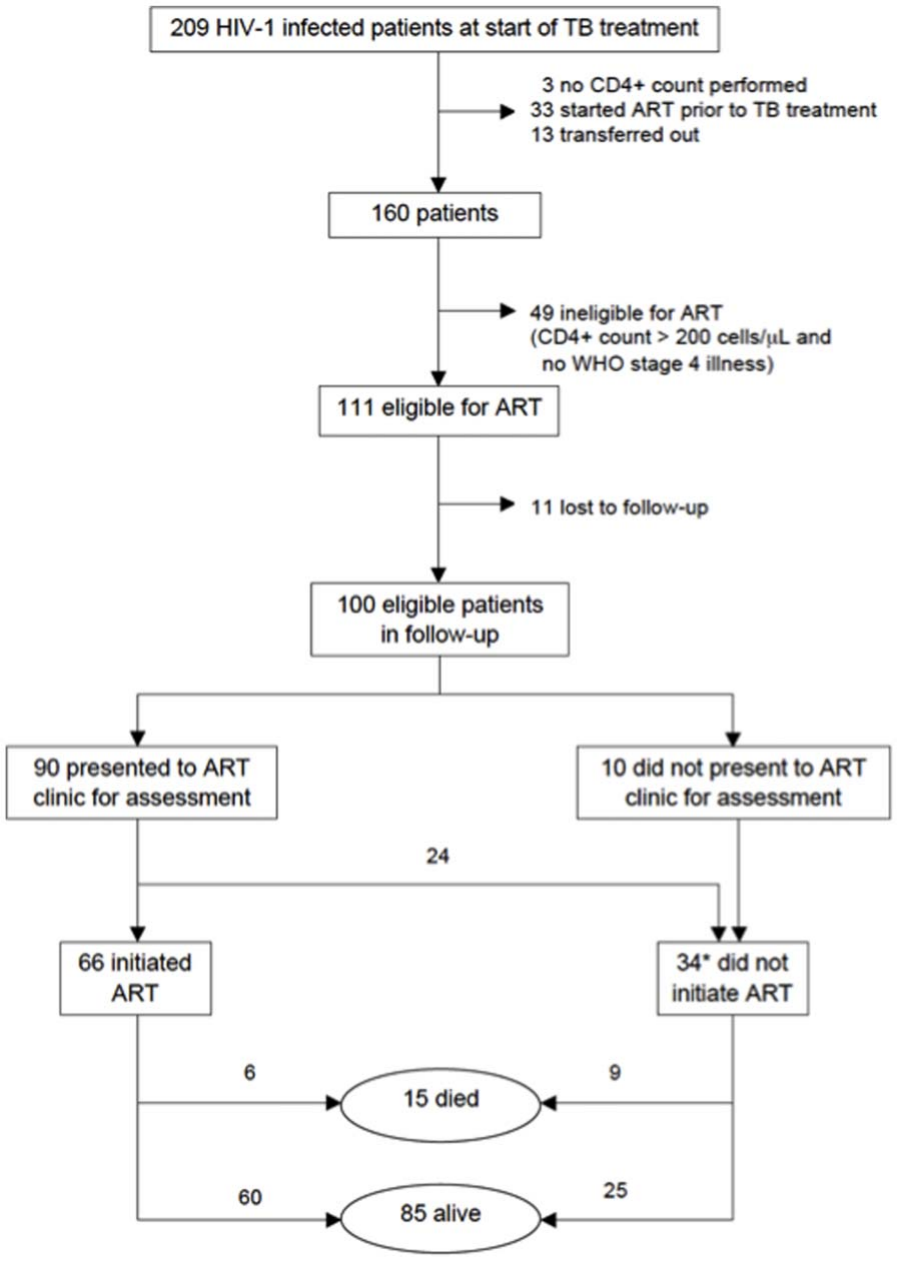
Flow-diagram showing inclusion criteria and outcomes of 100 eligible patients. ART: antiretroviral treatment, TB: tuberculosis, WHO: World Health Organisation, *11 of these 34 patients initiated antiretroviral treatment 4 to 91 days after completion of TB treatment.

The following is a secondary analysis of this cohort: among those eligible to receive ART, we determined factors associated with not initiating ART. Of 209 enrolled HIV-infected TB patients (figure 1), 100 comprised our study population as they were eligible to initiate ART at TB diagnosis, according to national guidelines. Reasons for excluding the remaining 109 TB patients are shown in figure 1: CD4+ count not performed (n = 3), ART started prior to TB treatment (n = 33), transferred out (n = 13), ineligible for ART as CD4+ count greater than 200 cells/µL (n = 49) and lost to follow-up (n = 11). We defined ‘transferred out’ as transfer of care to another tuberculosis clinic at a patient's request. This transfer was facilitated by a written referral letter and resulted in exclusion from our study. We defined ‘lost to follow-up’ as being unable to trace a TB patient 6 months after commencing TB treatment. We used clinic and hospital charts, as well as the Provincial Government of the Western Cape's electronic tuberculosis register (ETR.net)[11] to trace TB patients and record clinical outcomes.

We determined the proportion of eligible TB patients that did not initiate ART during TB treatment. We also determined those at greatest risk of i) not presenting to the ART clinic for assessment, ii) not initiating ART, and iii) death. In our setting, eligible TB patients were referred with a written letter to their nearest ART clinic for assessment. TB patients who presented to the ART clinic for assessment received ART education from trained counsellors. After appropriate counselling and evaluation by either a nurse or doctor, TB patients initiated ART. In our study, a TB patient who attended one or more ART clinic appointments was considered to have ‘presented to an ART clinic for assessment.’ We reviewed TB patients' hospital and ART charts, the Western Cape's electronic tuberculosis register (ETR.net)[11] and Cape Town's electronic *eKapa* ART database to record those who presented to an ART clinic for assessment, as well as those who initiated ART.

We defined clinical deterioration as symptomatic worsening or failure to stabilise within 24 weeks following initiation of TB treatment[8]. Causes of clinical deterioration included AIDS-defining illnesses (according to WHO stage 4 criteria), non AIDS-defining HIV-related infections, TB-related illnesses, and illnesses unrelated to TB, i.e. co-morbid illnesses. These illnesses have been described in detail in a previous report[8].

### Statistical analysis

We performed statistical analysis using Stata 10.0 (Texas, USA). We found that age, weight at TB diagnosis and duration to ART initiation were right (non-normal) skewed but had normal distributions with logarithmic transformation (Shapiro-Wilk test); we described these variables using means and 95% confidence intervals (95% CIs). The mean age was used to dichotomise age categories (age <36 years vs age ≥36 years). Proportions were calculated for categorical variables and described using 95% CIs. We used the Fisher exact test to determine which categorical variables were significantly associated with i) not presenting to an ART clinic for assessment, ii) not initiating ART and iii) death. A p-value of less than 0.05 was considered significant.

Using logistic regression analysis, we explored relationships between categorical variables and i) not presenting to an ART clinic for assessment, ii) not initiating ART and iii) death. Backward stepwise logistic models were proposed to quantify these relationships; these models were reported using adjusted odds ratios and 95% CIs. We fitted each model using the likelihood ratio, which was logarithmically transformed to generate the chi-squared statistic.

## Results

### Description of eligible adults in follow-up

The following features characterised the 100 eligible TB patients at TB diagnosis (table 1): The mean age was 36 years (95% CI: 23–57 years) and 54% (95% CI: 44–64%) were male. Men were older than women (mean age in years, 95%CI: 37 [23–59] vs. 35 [22–55]) but this difference was not statistically significant. At TB diagnosis, 70% of TB patients had a CD4+ count less than 100 cells/µL, 30% had a previous history of tuberculosis, 56% had no evidence of extra-pulmonary tuberculosis and 23% had results for TB drug susceptibility testing – most of whom were drug susceptible (87%, 20/23). During TB treatment, 95% of TB patients received trimethoprim-sulfamethoxazole chemoprophylaxis, 66% experienced clinical deterioration and 50% required hospital admission. At 6 months of TB therapy, drug sensitive *Mycobacterium tuberculosis* was cultured in 47 (88%) of 53 TB patients for whom drug susceptibility testing was performed.

**Table 1:** Baseline characteristics and microbiologic confirmation of tuberculosis in 100 HIV-1 infected patients eligible for ART and receiving TB treatment.

Male gender, n (%)	54	(54)
Age <36 years, n (%)	55	(55)
CD4+ count <100 cells/µL	70	(70)
TMP-SMX chemoprophylaxis, n (%)	95	(95)
Previous tuberculosis, n (%)	30	(30)
Diagnosis of TB at hospital, n (%)	55	(55)
No extra-pulmonary tuberculosis, n (%)	56	(56)
Drug susceptibility test results known at TB diagnosis, n (%)	23	(23)
Drug sensitive *M.tb* known at TB diagnosis, n (%)	20	(20)
Drug susceptibility test results known at 6 months, n (%)	53	(53)
Drug sensitive *M.tb* at 6 months, n (%)	47	(47)
Weight less than 50 kilograms (%)	30	(30)
Experienced clinical deterioration, n (%)	66	(66)
Admission to hospital, n (%)	50	(50)

ART: antiretroviral treatment, TB: tuberculosis, WHO: World Health Organisation.

ART: antiretroviral treatment, TB: tuberculosis, TMP-SMX chemoprophylaxis: daily trimethoprim-sulfamethoxazole chemoprophylaxis 160/800 mg, *M.tb: Mycobacterium tuberculosis*.

### Barriers to presentation to the ART clinic for assessment

Among the 100 eligible TB patients (figure 1), 90% presented to an ART clinic for assessment, while 10% did not. In univariate analyses (data not shown), the following were significantly associated with not presenting at an ART clinic for assessment: previous tuberculosis and admission to hospital. Using our logistic regression model, we found that previous TB remained significant (OR = 4.1, 95%CI: 1.04–16.42; Model P = 0.015, R^2^ = 0.129).

### Barriers to ART initiation

Of the 100 eligible TB patients, 66% (95%CI: 57–75%) initiated ART during TB treatment while 34% (95%CI: 25–43%) did not (figure 1). The mean interval from commencing TB treatment to ART initiation was 58 days (95%CI: 18–184 days). Of the 25 TB patients alive and not receiving ART at 6 months of follow-up, 11 (44%) initiated ART 4–91 days after completion of TB treatment. A high proportion of TB patients (24/90, 27%) did not initiate ART during TB treatment despite referral and presentation at an ART clinic for assessment.

In univariate analyses (table 2), the following were significantly associated with not initiating ART during TB treatment: male gender, younger age (<36 years), history of previous TB, absence of extra-pulmonary TB, and knowing the results of drug susceptibility testing at TB diagnosis. Using our logistic regression model (table 2), we found that male gender (OR = 3.7, 95%CI: 1.25–10.95) and younger age (OR = 3.3, 95%CI: 1.12–9.69) remained statistically significant (Model P = 0.003, R^2^ = 0.224). The odds of not initiating ART was 12 times greater in a young man (<36 years) as compared to an older woman (≥36 years). Forty-six percent (15/28) of eligible young men did not initiate ART as compared to 5% (1/19) of eligible older women.

**Table 2:** Univariate analysis and logistic regression model showing factors associated with not initiating ART during TB treatment.

	No ART (n = 34)		ART (n = 66)		P-value	aOR	(95%CI)	P-value
Male gender, n (%)	24	(71)	30	(45)	0.017*	3.7	(1.25–10.95)	0.018*
Age <36 years, n (%)	24	(71)	31	(47)	0.025*	3.3	(1.12–9.69)	0.031*
CD4+ count >100 cells/µL, n (%)	14	(41)	16	(24)	0.080	1.6	(0.54–4.87)	0.384
No TMP-SMX chemoprophylaxis, n (%)	3	(9)	2	(3)	0.208	2.4	(0.84–7.08)	0.102
Previous tuberculosis, n (%)	16	(47)	14	(21)	0.008*	3.2	(0.33–31.68)	0.311
Diagnosis of TB at clinic, n (%)	21	(62)	33	(50)	0.264	1.9	(0.47–7.32)	0.378
No extra-pulmonary tuberculosis, n (%)	24	(71)	32	(48)	0.035*	2.3	(0.73–7.53)	0.151
Drug susceptibility test results known at TB diagnosis, n (%)	13	(38)	13	(20)	0.045*	2.1	(0.67–6.39)	0.209
Weight less than 50 kilograms	13	(38)	17	(26)	0.214	1.1	(0.35–3.61)	0.851
No clinical deterioration, n (%)	14	(41)	20	(30)	0.277	2.2	(0.73–6.86)	0.158
Admission to hospital, n (%)	18	(53)	32	(48)	0.673	2.6	(0.70–9.50)	0.155

*P<0.05 considered statistically significant, Model likelihood ratio: P = 0.003, R^2^ = 0.2235.

aOR: adjusted odds ratio, 95% CI: 95% confidence interval, ART: antiretroviral treatment, TB: tuberculosis, TMP-SMX chemoprophylaxis: daily trimethoprim- sulfamethoxazole chemoprophylaxis 160/800 mg.

### Mortality

Fifteen TB patients died during TB treatment. In univariate analyses (table 3), the following were significantly associated with death during TB treatment: younger age (<36 years), CD4+ count <100 cells/µL, not presenting to an ART clinic for assessment, not initiating ART, experiencing clinical deterioration, and admission to hospital. Using our logistic regression model (table 3), only a CD4+ count <100 cells/µL remained statistically significant (OR = 18.0, 95%CI: 1.55–210.62; Model P = 0.012, R^2^ = 0.287).

**Table 3:** Univariate analysis and logistic regression model showing factors associated with death during TB treatment.

	Died (n = 15)		Alive (n = 85)		P-value	aOR	(95%CI)	P-value
Male gender, n (%)	11	(73)	43	(51)	0.103	4.0	(0.77–20.77)	0.099
Age <36 years, n (%)	12	(80)	43	(51)	0.035*	3.8	(0.76–18.94)	0.103
CD4+ count <100 cells/µL, n(%)	14	(93)	56	(66)	0.032*	18.0	(1.55–210.62)	0.021*
No TMP-SMX chemoprophylaxis, n (%)	2	(13)	3	(4)	0.108	15.8	(0.49–506.22)	0.118
Previous tuberculosis, n (%)	5	(33)	25	(29)	0.760	3.5	(0.57–21.21)	0.177
Diagnosis of TB at hospital, n (%)	9	(60)	41	(48)	0.401	1.2	(0.25–5.39)	0.839
No extra-pulmonary tuberculosis, n (%)	8	(53)	48	(56)	0.822	1.5	(0.31–6.89)	0.631
Drug susceptibility test results known at TB diagnosis, n (%)	6	(40)	20	(24)	0.180	1.3	(0.28–6.39)	0.724
Weight less than 50 kilograms (%)	6	(40)	24	(29)	0.375	1.1	(0.35–3.61)	0.851
Did not present to ART clinic for assessment	4	(27)	6	(7)	0.012*	2.2	0.29–16.27	0.448
ART not initiated, n (%)	9	(60)	25	(29)	0.021*	3.6	(0.70–18.36)	0.124
Experienced clinical deterioration, n (%)**	15	(100)	51	(60)	0.047*			
Admission to hospital, n (%)**	15	(100)	35	(41)	0.006*			

*P<0.05 considered statistically significant;

**both the following variables were collinear, and omitted from the model: admission to hospital and experienced clinical deterioration; Model likelihood ratio: P = 0.012, R^2^ = 0.287; aOR: adjusted odds ratio, 95% CI: 95%. confidence interval, ART: antiretroviral treatment, TB: tuberculosis, TMP-SMX chemoprophylaxis: daily trimethoprim- sulfamethoxazole chemoprophylaxis 160/800 mg.

### Estimated burden due to increase in CD4+ threshold from 200 to 350 cells/µL

**Figure 2:**
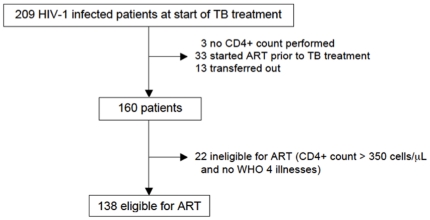
Flow-diagram showing eligible patients according to new South African antiretroviral treatment guidelines [**25**].

When the threshold CD4+ count for ART initiation is adjusted from less than 200 cells/µL to less than 350 cells/µL, according to the new South African guidelines that were implememented 6 months after completion of this study, the number of eligible TB patients requiring ART increases by 24% (95% CI: 16–32%) – from 111 to 138 patients (figure 2).

## Discussion

Timely initiation of ART in eligible HIV-infected TB patients is a priority in South Africa. Knowing which barriers prevent ART initiation, as well as the treatment gap among eligible TB patients, is invaluable information for TB and HIV policy-makers who facilitate ART provision. In this study, we found that one-third of eligible TB patients did not initiate ART during TB treatment.

Our rate of ART initiation (66% during the first 6 months after initiating TB treatment) is higher than reported elsewhere in South Africa [12] – we attribute this to the close physical proximity of our ART and TB clinics. Over the past few years, interventions at our facility have integrated ART and TB services [13]. These interventions include high rates of voluntary counseling and testing of HIV status, testing of CD4+ counts at HIV diagnosis, and expedited referral to ART services. We found that one-third of eligible patients did not initiate ART during TB treatment, despite the close proximity of our TB and ART services. We believe that our ART initiation rates, while laudable, could be further improved.

In our study, younger age and male gender increased the odds of not initiating ART during TB treatment. To our knowledge, no studies have reported this significant association. Previous studies have mostly reported findings from HIV cohorts, where it has been shown that male gender is associated with substantial problems during ART. A Malawian study found that male gender and a WHO stage 4 disease increased the risk of early death during ART [14]. Similarly, Cornell *et al* found that men experienced higher early mortality on ART compared to women, largely due to their presentation with more advanced HIV disease [15]. Recently, a Nigerian study reported that death is significantly associated with younger age and male gender, independent of a low CD4+ count[16] – risk factors for not initiating ART, however, were not investigated. While men are at higher risk than women to default ART [17], one study reported no substantial sex differences in the *benefits* of ART [18]. In our study, while many presented to the ART clinic for assessment, young men were at greatest risk of not initiating ART. It appears that these young men missed an ideal opportunity to initiate ART. Reasons for this failure need to be determined and addressed. Possible explanations include apathy[19], social stigma, lack of insight, lower socio-economic status and poor social support networks. An alternate explanation is that initiation of ART is more socially acceptable among women and older men. Further studies are needed to elucidate which of these factors prevent young adult men from initiating ART during TB treatment.

Our study has a number of strengths. Firstly, our cohort is comparable to other TB cohorts in South Africa: these cohorts share similar demographic profiles[8], [20], socio-economic status[9], rates of voluntary counselling and testing for HIV-1 infection (>80%)[8,personal communication – Judy Caldwell, Cape Town Health Department], rates of HIV/TB co-infection (>70%)[8], [21], distribution of CD4+ counts at TB diagnosis (CD4+ counts from 1-200, 201–350 and >350 cells/µL of 64%, 18% and 17%, respectively)[8, personal communication – Judy Caldwell, Cape Town Health Department], and proportion of HIV-1 infected patients with TB that die (15%)[8], [22]. Secondly, in this study, we recorded a number of unique variables not routinely available in electronic TB registers in South Africa, which we incorporated in our logistic regression models. These unique variables included drug susceptibility results, occurrence of clinical deterioration, presentation at the ART clinic for assessment, initiation of ART and admission to hospital during treatment.

We also found that TB patients with a history of previous TB were less likely to present to an ART clinic for assessment. This finding is disconcerting, especially as these patients presented to health-care facilities on a daily basis for streptomycin injections, and could have been referred to ART services by health-care providers. To overcome this problem, ART counselling should be offered to all HIV-infected TB patients at each clinic visit. We also found that a CD4+ count less than 100 cells/µL was the only significant risk factor for death, regardless of whether ART was initiated or not. This recapitulates the importance of ART initiation in patients with tuberculosis, prior to severe immune-suppression.

We recognise certain limitations in this study. The proportion of eligible patients referred to ART clinics was not ascertained. However, we observed that >95% of adults knew their HIV status[8], ∼99% of HIV-infected TB adults had CD4+ counts performed at TB diagnosis, and 90% of eligible TB patients presented to ART clinics for assessment. We were unable to determine whether early or late ART during TB treatment is more beneficial, and await the findings of larger trials to answer this question[23]. We acknowledge that in other parts of Africa, where greater geographical distances exist between TB and ART clinics, obstacles to ART initiation may differ[24]. We note that 11 eligible TB patients were not included in our analyses as they were lost to follow-up. If these 11 TB patients are included, the proportion that did not initiate ART increases from 34% (34/100) to 41% (45/111). We also acknowledge that we were unable to address other socio-demographic barriers as these data were not collected prospectively.

Finally, with the recent change in national guidelines to increase the CD4+ count threshold for ART [25], we anticipate a substantial increase (by 24%) in the number of eligible TB patients requiring ART.

### Conclusion

In a clinic with integrated tuberculosis and HIV services, one-third of eligible TB patients – particularly young men – did not initiate ART. Research is needed to determine why eligible young men are at greatest risk of not initiating ART. It is our hope that further characterisation of these factors will result in strategies to increase ART initiation during TB therapy.

### Disclaimer

The contents of this article are the responsibility of the authors and do not necessarily reflect the views of the US Agency for International Development or the US government.
